# Post-Translational Modifications of Histones in Vertebrate Neurogenesis

**DOI:** 10.3389/fnins.2015.00483

**Published:** 2015-12-24

**Authors:** Nikolaos Mitrousis, Vincent Tropepe, Ola Hermanson

**Affiliations:** ^1^Institute of Biomaterials and Biomedical Engineering, University of TorontoToronto, ON, Canada; ^2^Department of Cell and Systems Biology, Centre for the Analysis of Genome Evolution and Function, University of TorontoToronto, ON, Canada; ^3^Department of Neuroscience, Karolinska InstitutetStockholm, Sweden

**Keywords:** histone PTMs, histone acetylation, histone methylation, chromatin, neurogenesis, neural stem cell

## Abstract

The process of neurogenesis, through which the entire nervous system of an organism is formed, has attracted immense scientific attention for decades. How can a single neural stem cell give rise to astrocytes, oligodendrocytes, and neurons? Furthermore, how is a neuron led to choose between the hundreds of different neuronal subtypes that the vertebrate CNS contains? Traditionally, niche signals and transcription factors have been on the spotlight. Recent research is increasingly demonstrating that the answer may partially lie in epigenetic regulation of gene expression. In this article, we comprehensively review the role of post-translational histone modifications in neurogenesis in both the embryonic and adult CNS.

## Introduction

Histone post-translational modifications (PTMs) have been implicated in a multitude of developmental processes and diseases (Bhaumik et al., 2007; Chi et al., 2010; Tyssowski et al., 2014; Yao and Jin, 2014). Throughout the last decade, interest into the role of histone PTMs in neurogenesis has risen. The goal of this review is to provide an overview of the current evidence for histone PTM involvement in neurogenesis, the known mechanisms that initiate these processes, as well as investigate the contribution of histone PTMs to the complex and interconnected network of epigenetic modifications. We begin with a brief introduction into neurogenesis and histone PTMs, then move on to discuss the major histone PTMs (i.e., acetylation and methylation) that have been investigated in the context of neurogenesis, highlighting a few studies that exemplify the main conceptual insight. Lastly, we discuss how these processes are initiated and how specificity is achieved.

### Neurogenesis

**Figure 1:**
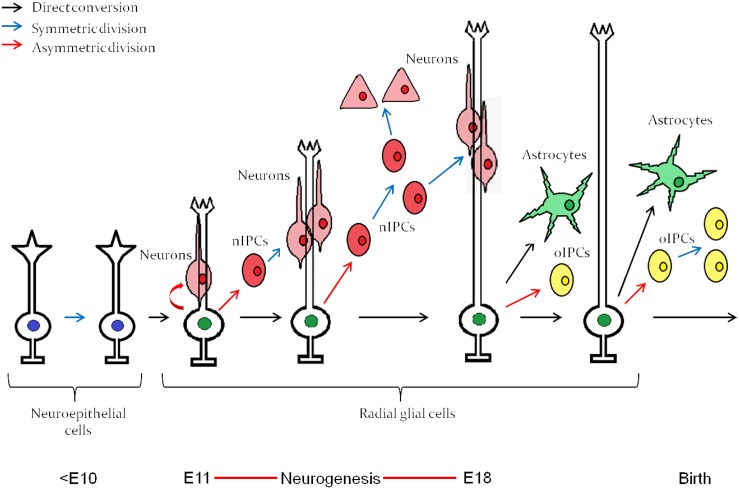
**Embryonic neuroepitethial (NE) cells of the neural tube give rise to radial glial cells, which through an intrinsic clock differentiate first into neurons and then into astrocytes**. The neurogenesis “window” extends approximately from E11 to E18. nIPC, neurogenic intermediate progenitor cell; oIPC, oligodendrogenic intermediate progenitor cell. Note that no oligodendrocytes are generated before birth. The figure represents embryonic neurogenesis in the mouse. Adapted from Kriegstein and Alvarez-Buylla (2009).

Neurogenesis in vertebrates begins after ectodermal cells acquire a neuroepithelial identity through the process of induction and subsequent morphological transformation to become radial “glial” cells with stem cell properties (Dang and Tropepe, 2006; Kriegstein and Alvarez-Buylla, 2009). Neuroepithelial cells, followed by radial glial cells, undergo a period of population expansion before radial glial cells differentiate into committed transit amplifying progenitors (Figure 1) that eventually give rise to mature cells of the central nervous system (CNS). Through a cell intrinsic clock, these neural stem cells initially differentiate into neurons and switch their competence toward generating astrocytes (Miller and Gauthier, 2007), as depicted in Figure 1. Oligodendrocytes are the last fate to arise and are typically generated during post-embryonic stages. Interestingly, isolated neural stem cells from the embryonic cerebral cortex follow the same order of differentiation *in vitro*, initially giving rise to neurons and then astrocytes (Qian et al., 2000). The mechanism by which this intrinsic clock is established, which is common among most developing neural tissues, has been a long lasting question in developmental neurobiology.

Neurogenesis persists in adulthood with notable species variation, but is typically limited to specific neurogenic regions that can be associated with functional plasticity and regeneration (Lindsey and Tropepe, 2006; Ming and Song, 2011; Kaslin et al., 2013). Although adult neural stem cells share most characteristics with their embryonic counterparts, such as marker expression and multipotentiality *in vitro* (Reynolds and Weiss, 1992; Weiss et al., 1996), they are typically restricted under physiological conditions producing primarily neurons *in vivo* (Alvarez-Buylla and Nottebohm, 1988; Grandel et al., 2006; Lledo et al., 2008). Thus, while the process of neurogenesis is fundamentally conserved among brain regions, ontogeny and putatively throughout vertebrate evolution, neurogenesis is nonetheless shaped by significant adaptations to distinct physiological states and life histories (Tropepe, 2008).

Factors that influence the extent of neurogenesis include proliferation and survival of neural stem and progenitor cells (NSPCs), their efficiency of differentiation into neurons and glial cells, and the survival and function of the differentiated progeny. The term NSPCs is used when neural stem cell identity cannot be readily distinguished from that of a more committed progenitor cell identity.

### Histone post-translational modifications

The nucleosome, a fundamental unit of chromatin, consists of 146–147 bp of DNA that is wrapped around 1 histone octamer, which includes 2 molecules of each of the core histones H2A, H2B, H3, and H4 (Kouzarides, 2007). In the last two decades it has become increasingly evident that nucleosomes have a broader role than just facilitating the packaging of DNA into the tiny space that is a cell nucleus. Histones participate in the regulation of gene expression and are the target of a plethora of transcription factors or associated proteins. The histone N-termini that protrude from the tightly packed octamer, are somewhat less structured (Kouzarides, 2007; Bannister and Kouzarides, 2011). They are free to interact with DNA, and are also exposed to modification enzymes. These enzymes modify histone N-termini by, for instance acetylation, methylation, phosphorylation, SUMOylation, ubiquitination, citrullination, or ribosylation (Tan et al., 2011). Each of these modifications has an effect on gene expression through 2 potential mechanisms. The first one is based on electrostatic interactions (Zentner and Henikoff, 2013). Specifically, DNA is negatively charged and histone N-termini are positively charged. Depending on the modification, the positive charge of the N-terminus may be concealed or exposed. For example, acetylation of a lysine residue will mask its positive charge and prevent strong attraction to the DNA. This will lead to a more relaxed chromatin state, and when this occurs in a promoter, transcription factors have more room to bind DNA and exert their functions. The converse would happen in the case of histone deacetylation at a lysine residue. The second major mechanism of action of chromatin modifications is through creating binding sites for transcription factors and adaptor proteins that recognize specifically modified histone residues (Bannister and Kouzarides, 2011; Zentner and Henikoff, 2013).

While histone PTM is a major contributor to epigenetic regulation of gene expression, it is important to keep in mind that it represents only one aspect of an ever-expanding network of epigenetic regulators (Yao and Jin, 2014). Epigenetics can be loosely defined as changes in gene expression, or the phenotype, that are not induced by changes in the DNA sequence (Bird, 2007). In addition to histone PTMs, a long list of regulatory mechanisms of gene expression fit under this term including DNA methylation, microRNAs, long non-coding RNAs and methyl-DNA binding proteins. In reality, the various epigenetic mechanisms co-operate and form complexes or groups of enzymes in which there is a cascade of signals linking an extracellular trigger to a gene expression event (Jobe et al., 2012).

## Histone PTMs in neurogenesis

### Histone acetylation

Histone acetylation was the first histone PTM to be discovered as well as associated with gene expression (Phillips, 1963; Allfrey et al., 1964). Histones are acetylated at lysine residues by histone acetyl-transferases (HATs) and are deacetylated by histone deacetylases (HDACs) (Bannister and Kouzarides, 2011). The involvement of histone acetylation in neurogenesis is widespread and ranges from embryonic neurogenesis, to adult neuronal survival and differentiation. Below we describe, using selected examples, how histone acetylation could influence the process of neurogenesis.

#### CREB/CBP and their roles in neurodevelopment and neurodegeneration

One of the initial culprits to be identified was c-AMP responsive element binding protein (CREB) (Montminy and Bilezikjian, 1987; Lonze and Ginty, 2002; Dworkin and Mantamadiotis, 2010). CREB is a generic transcriptional activator that is involved in a plethora of developmental processes as well as cancer, and it functions by recruiting CREB-binding protein (CBP or CREBBP), which possesses HAT activity (Chrivia et al., 1993). CREB function has been implicated in neuronal plasticity, hippocampal learning and memory (Bailey et al., 1996), partially by regulating the secretion of the growth factor BDNF (Tao et al., 1998). Various reports have also shown that CREB is involved in both embryonic and adult neurogenesis (Young et al., 1999; Zhu et al., 2004). CREB knockout mice (Dworkin et al., 2009) analyzed at E14.5 display severe deformities in both their embryonic brain and retina, sometimes completely lacking an olfactory bulb. Additionally, embryonic NSPCs from CREB^−∕−^ mice show decreased survival *in vitro*, but normal differentiation (Dworkin et al., 2009). Investigating the mechanism, the authors identified that the transcripts for the anti-apoptotic protein bcl-2 and the growth factors BDNF, NGF and PACAP were decreased in CREB ^−∕−^ NSPC cultures. Interestingly, analyzing CREB^−∕−^ mice at E18.5, Rudolph et al. (1998) reported only subtle morphological alterations of the brain structure. This indicates that CREB loss of function could delay the neurogenesis “window” so that obvious deformities exist at early stages but might be compensated for at later stages in development. An alternative explanation for these seemingly conflicting results could be based on the cAMP response element regulatory protein (CREM). CREM belongs to the CREB family of transcription factors and is known to compensate for the lack of CREB (Hummler et al., 1994; Mantamadiotis et al., 2002). In the report by Rudolph et al., there was a strong upregulation of CREM upon CREB knockout at E18.5, which was not observed by Dworkin et al. at E14.5. This, along with observations in very early CREB^−∕−^ embryos (Bleckmann et al., 2002) may suggest that the complementation of CREB function by CREM may only occur at time points later than E14.5. In order to overcome this functional redundancy issue, Mantamadiotis et al. (2002) generated conditional CREB knockout mice on a CREM^−∕−^ background. When the deletion was driven by Nestin-Cre, therefore occurring in NSPCs, there was a decrease in brain size with differences becoming obvious from E16.5 onwards, which coincided with the onset of strong CREB deletion. The cortex and the hippocampus were the most strongly affected areas, and mice died perinatally. The phenotype was attributed to increased apoptosis in the affected areas. Interestingly, when CREB deletion was driven by the promoter of calcium/calmodulin-dependent protein kinase II-α (CamkIIα) gene to drive expression in mature neurons postnatally, a progressive neuronal degeneration over the course of 6 months was observed. The areas mostly affected were the hippocampus and the striatum and the animals exhibited a behavioral phenotype reminiscent of models of neurodegenerative disease (Lalonde, 1987; Mangiarini et al., 1996; van den Akker et al., 1999; Dragatsis et al., 2000; Yamada et al., 2001). Therefore, these studies demonstrated that histone acetylation triggered by CREB is necessary for neurogenesis both at the NSPC and the mature neuron level.

Notably, histone acetylation has also been implicated in neurodevelopmental disorders. Rubinstein-Taybi syndrome is characterized by cognitive dysfunction, and caused by a haploinsufficiency in the *cbp* (encoding for CBP) gene (Roelfsema and Peters, 2007). Cbp^+∕−^ mice show cognitive impairment in adulthood, which gave rise to the hypothesis that the effect is due to defective neural circuits (Josselyn, 2005). Wang et al. (2010) demonstrated that cbp^+∕−^ mice also show deficits in embryonic neurogenesis and gliogenesis. Cbp^+∕−^ pups displayed an altered frequency, duration and number of ultra-sonic vocalizations (USV) after separation from their mothers. USV changes are believed to reflect cognitive and social behavior impairments (Branchi et al., 2001). Embryonic neural precursors differentiated into all three lineages (neurons, astrocytes, oligodendrocytes) with less efficiency both *in vitro* and *in vivo*, when CBP was knocked down. The HAT activity of CBP was necessary for that effect, since an HDAC inhibitor could rescue the effects of the CBP knockdown. CBP was shown to directly bind and acetylate the H3K9/14 at promoters of genes involved in neural (βIII-tubulin), astrocytic (GFAP, S100β) and oligodendrocytic (MBP, PLP2) development, thus up-regulating their expression. Moreover, the accessibility of HDACs and CBP to gene promoters involved in NSPC specification in the zebrafish hindbrain is regulated by the interaction of Meis and Pbx transcription factors (Choe et al., 2009). This indicates that transcription factors can act as gatekeepers, or conversely molecular beacons, for HDAC/HAT recruitment in a region specific manner. Lastly, the phosphorylation of CBP by atypical protein kinase C ζ (aPKCζ) was shown to be necessary for promoting the differentiation of neural precursors, and has been linked to spatial memory formation (Wang et al., 2010, 2012). The latter provides a functional link between CBP function and extracellular signals that are known to be involved in neurogenesis, such as fibroblast growth factors (FGFs) and neurotrophins (Kengaku and Okamoto, 1993; Bartkowska et al., 2007), and may pave the way to new approaches for treatment of various neurological disorders.

#### Histone acetylation and neuronal differentiation: the case of orexin neurons

**Figure 2:**
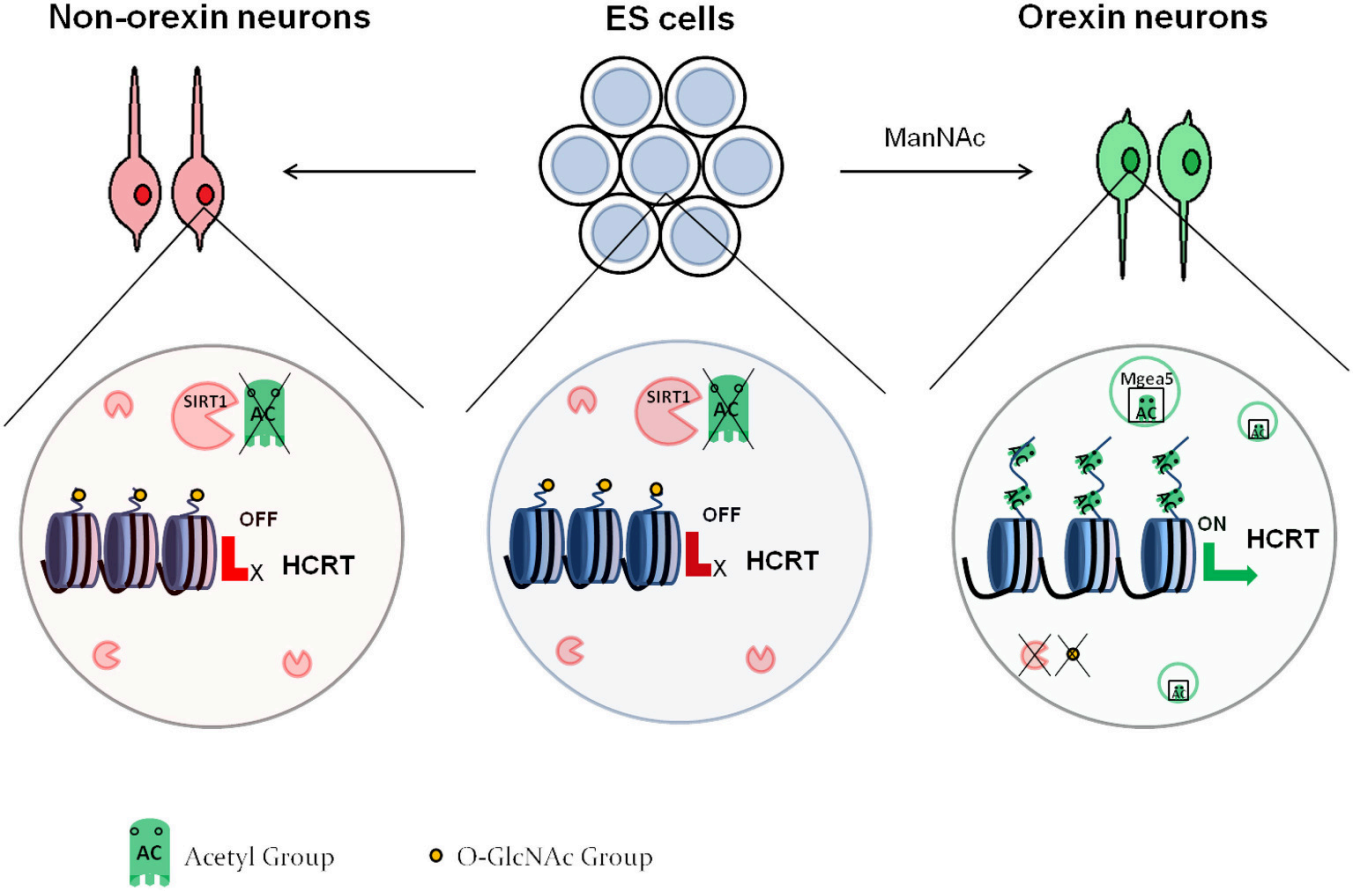
**Epigenetic regulation of orexin neuron differentiation**. In embryonic stem cells as well as non-orexin neurons, SIRT1 deacetylates the histones at the regulatory region of the HCRT gene, leading to repression of its expression. These histones also contain O-GlcNAc residues. Treatment with ManNAc activates Mgea5, which acetylates the histones of the HCRT regulatory region, and leads to removal of the GlcNAc residues. This activates the expression of the HCRT gene, and the cells turn into orexin neurons.

Histone acetylation appears to also be involved in the differentiation of specific neuronal subtypes. Hayakawa et al. (2013) showed that the differentiation of orexin neurons from mouse embryonic stem (ES) cells depends on DNA methylation and histone acetylation. Orexin neurons are localized in the hypothalamus and are involved in the sleep/wake cycles as well as feeding behavior. Orexin neuron differentiation requires the expression of hypocretin (orexin) neuropeptide precursor (Hcrt), which contains two proximal regulatory regions that are CpG island-rich. The authors demonstrated that mES cells, as well as neurons derived from mES cells under standard differentiation conditions had high DNA methylation at these regions, as well as low histone acetylation at various lysine residues, which translates to low gene expression. On the contrary, treatment with *N*-acetyl-*D*-mannosamine (ManNAc) induced an increase in histone acetylation at all of the assessed sites. The authors proceeded to pinpoint that Sirt1, a class III HDAC, inhibited orexin neuron differentiation. They finally demonstrated that meningioma-expressed antigen 5 (Mgea5) was necessary for the effect of ManNAc into orexin neuron differentiation. Mgea5 has a dual enzymatic activity: it is a HAT and also serves as an O-GlcNAcase, which removes *O*-linked *N*-acetyl glycosamine (O-GlcNAc) residues from proteins including histones. Interestingly, both enzymatic activities were required for orexin neuron differentiation. This observation led to a model (Figure 2), whereby in non-orexin neurons, the Hcrt regulatory regions have deacetylated histones and are O-GlcNAc rich. In orexin neurons, Mgea5 expression leads to removal of the O-GlcNAc residues and acetylation of core histones, which induces the expression of Hcrt. The substrate of the O-GlcNAcylation remains to be defined, even though core histones are a probable candidate (Sakabe et al., 2010; Fujiki et al., 2011; Fong et al., 2012).

#### Region and cell-type specific differences in neurogenesis

The histone code of neurogenesis is not universal. Different areas of the central nervous system can be differentially affected by the same histone modification. Shakèd et al. (2008) demonstrated that HDACs of classes I and II are necessary for neurogenesis in the embryonic ganglionic eminence, but inhibit neurogenesis in the embryonic cortex. The results held true both *in vitro* and *in vivo*. The authors used Trichostatin A (TSA), a chemical inhibitor of class I and II HDACs (Yoshida et al., 1990). The mechanism of the observed effect was shown to involve the BMP2/4 pathway, which induces neurogenesis in the cortex but promotes astrogliogenesis in other areas of the brain (Gross et al., 1996; Li et al., 1998; Lim et al., 2000). In studies assessing the effects of HDAC inhibition on mature neuronal survival, Forgione et al. (Forgione and Tropepe, 2011, 2012) found that treatment of cells isolated from the embryonic mouse ventral midbrain with the HDAC inhibitors TSA, valproic acid (VPA) or sodium butyrate (SB) induced cell death in neurons but not astrocytes and the cell death was largely caspase-independent. Intriguingly, the same treatment did not result in neuronal cell death in cultures from the embryonic cortex. These studies underline the complexity of the epigenetic regulatory network of neurogenesis and indicate that a one-size-fits-all model might not be entirely correct. We will describe and discuss some of these aspects in the following sections.

#### Lessons learned from HDAC inhibitor experiments

Chemical inhibitors of global HDAC function such as TSA, VPA and SB have been used to investigate the role of histone acetylation in neurogenesis and yielded somewhat conflicting results.

Hsieh et al. assessed the levels of acetylated histones H3 and H4 during adult rat hippocampal NSPC differentiation into neurons, astrocytes and oligodendrocytes and found that neuronal differentiation was associated with a greater maintenance of acetyl-H3 and acetyl-H4 than differentiation into the other 2 lineages (Hsieh et al., 2004). They proceeded to show that treatment with VPA increased neuronal differentiation of these NSPCs at the expense of glial differentiation, and confirmed the results using the HDAC inhibitors TSA and SB. The effect was attributed to induction of NeuroD1, a known transcription factor involved in neurogenesis (Miyata et al., 1999), by the VPA treatment. Intriguingly, VPA treatment led to 3 times as much cell death as in control cultures, which implies that selective survival of neuronal progenitor cells could also be involved. VPA treatment *in vivo* also decreased BrdU incorporation and increased neuronal differentiation in the hippocampus of adult rats, which supports the *in vitro* observations. Corroborating the study of Hsieh et al. (Yu et al., 2009) also observed decreased BrdU incorporation in hippocampal neural stem cells after VPA treatment, and increased differentiation both *in vitro* and *in vivo*.

On the contrary, Hao et al. (2004) showed that VPA treatment of adult mice led to an increase in BrdU incorporation in the hippocampus. Furthermore, Kim et al. (2009) found that administration of the HDAC inhibitors SB or TSA after ischemic hypoxia increased the BrdU incorporation and the Ki67 staining in the forebrain neurogenic zones of adult rats. In addition, PSA-NCAM staining was augmented with the treatments, which indicates that HDAC inhibition resulted in an increase in the presence of neuronal progenitors and/or immature neurons. An increase in the number of BrdU+ cells typically indicates increased proliferation of the NSPCs. However, the state of the cell cycle of NSPCs is an important consideration when interpreting BrdU incorporation data. For example, an *in vitro* study by Zhou et al. (2011) demonstrated that neural stem cells from the forebrain subependymal zone of adult mice exhibited a G1-to-S cell cycle arrest upon treatment with SB or suberoylanilide hydroxamic acid (SAHA), another HDAC inhibitor. If cells treated with HDAC inhibitors are gradually prolonging their cell cycle toward eventual cell cycle exit, then there could be a period of increased numbers of BrdU+ cells before they undergo differentiation or cell death. The study by Zhou et al. (2011) showed that neuronal specification genes such as NeuroD1, Neurogenin 1 and cell cycle inhibitors such as p21 and p27 were up-regulated with the treatments while progenitor associated genes, such as Sox2 and the Notch effectors Hes1 and Hes5 were down-regulated, suggesting that these cells prematurely differentiated into neurons.

In general, these studies suggest that chemically inhibiting HDACs may alter the behavior of NSPCs in the adult brain. In some cases this could lead to a prolonging of the cell cycle prior to exit or it could lead to premature cell cycle exit and this could depend on the type NSPC or other factors. Ultimately, HDAC inhibition seems to facilitate neuronal differentiation from progenitor cells, but also cause cell death if the inhibition is prolonged after neurons have differentiated. One caveat is the potential for off-target effects and toxicity when using chemical inhibitors (Marks et al., 2003; Wang et al., 2009; Forgione and Tropepe, 2011; Sun et al., 2011). Additionally, HDACs are enzymes of broad specificity that often target and remove acetyl-groups from transcription factors and other non-histone proteins. Therefore, the observed phenotype could be a result of these non-histone effects, which makes the data interpretation difficult.

Another explanation may be cell-specific expression of HDACs. Spatial and temporal variations in levels of expression of individual HDACs in different cell types and/or different stages of development should affect the genome wide occupancy of the HDACs. A pan-inhibition of several types of HDACs could therefore have different and even opposite effects in progenitors from different regions and at different stages of maturation. It is therefore important to elucidate roles for specific HDACs *in vivo* and *in vitro*.

#### Lessons learned from experiments using knockdowns of specific HDACs

Genetic knockout studies have enabled us to further decipher the role of specific HDACs, as well as interactions between different HDACs, in regulating neurogenesis. Conditional knockouts of HDAC1 and HDAC2 driven by GFAP-cre, which is expressed in neural stem cells and astrocytes, revealed that their functions are redundant (Montgomery et al., 2009). When either HDAC1 or HDAC2 were missing, no obvious phenotype was observed. The double knockout mice, in contrast, showed severe structural abnormalities in the cortex, hippocampus and cerebellum and died within the first postnatal week. When E14.5 cortical NSPCs were cultured *in vitro*, the double knockout cells exhibited an almost complete blockade of neuronal differentiation, while the astrocytic differentiation was unaffected. The phenotype was attributed to increased apoptosis of neuronal progenitors both *in vitro* and *in vivo*, but could also be explained by a lack of maintenance of the differentiated neuronal state in the absence of HDAC1/2 function consistent with the effects of HDAC inhibitors on relatively mature neurons *in vitro* (Forgione and Tropepe, 2011). Therefore, this report unequivocally demonstrated that HDAC1 and HDAC2 are necessary for proper embryonic neurogenesis, and is in agreement with results obtained using dominant negative HDAC1 or HDAC2 (Humphrey et al., 2008). In addition, specific roles for HDAC2 in oligodendrocyte differentiation and HDAC3 in neuronal differentiation linked to acetylation of lysine 14 visavi 9 on histone H3 (H3K14, H3K9) were suggested based on RNA knockdown studies in rat embryonic cortical progenitors *in vitro* (Castelo-Branco et al., 2014). An important lesson from all of these studies is that global inhibition of HDACs can mask the contributions of individual HDACs to neurogenesis leading to conflicting observations especially when comparing different experimental contexts. Nonetheless, it is equally apparent that the regulation of histone deacetylation occurs at multiple levels and includes the specific subtype of HDAC, the specific cell type involved, as well as the region of the brain in which the deacetylation is occurring. The intersections between these complex levels of regulation no doubt enable the fine-tuning of neurogenesis, yet the details of these processes remain obscure.

### Histone methylation

In addition to histone acetylation, another PTM that was discovered in the 1960's is histone methylation (Allfrey et al., 1964). Histone methylation used to be considered an irreversible mark, in stark opposition to the dynamic nature of histone acetylation, but the discovery of histone demethylases has altered that view (Shi et al., 2004; Cloos et al., 2008; Bannister and Kouzarides, 2011). Histone methylation can occur at lysine or arginine residues. Lysine methylation is catalyzed by histone methyl-transferases (HMT), and lysines can be mono-, di- or tri-methylated. The number of methyl-groups added can alter the resulting phenotype. Arginine methylation is catalyzed by protein arginine methyl-transferases (PRMT), and can lead to mono-methylated, symmetrically di-methylated, or asymmetrically di-methylated arginines. Unlike histone acetylation, the effects that methylation has on gene expression depend on the specific residue that gets modified. For example, trimethylation of the lysine 4 residue of histone 3 (H3K4) is considered a mark of transcriptional activation, while trimethylation of H3K27 is a mark of transcriptional repression (Schuettengruber et al., 2007). There is substantial evidence for the involvement of histone methylation in neurogenesis.

#### The action of methyltransferases on embryonic neurogenesis

As mentioned in the introduction, during brain development neural stem cells initially give rise to neurons, and at about E18 in the mouse there occurs a switch in differentiation competence toward astrogliogenesis. Probing the mechanism of this intrinsic switch, Tan et al. (2012) demonstrated that ESET, an H3K9 methyltransferase previously known to be required for the maintenance of the pluripotent state in ES cells (Bilodeau et al., 2009; Yeap et al., 2009; Yuan et al., 2009), is pivotal for the regulation of the fate competence of NSPCs during development. Conditional knockout of ESET in the embryonic forebrain unveiled an intriguing finding: ESET seemed to be required for the generation of the deep cortical cell lineages that arise first, but the effects on later superficial cortical cell lineages was less pronounced. Additionally, the knockout of ESET led to precocious astrogliogenesis, demonstrated by markedly increased GFAP staining both *in vivo* and in NSPCs *in vitro* (Tan et al., 2012). The converse experiment of over-expressing ESET by *in utero* electroporation showed reduced astrocytic differentiation. In assessing the mechanism of action of ESET, the authors demonstrated by ChIP that it directly binds the promoters of GFAP and Sox9, associated with astrogliogenesis, repressing their expression by H3K9 tri-methylation. In the absence of ESET, these genes exhibit less of the H3K9me3 mark, and are over-expressed. On the contrary, many genes associated with neurogenesis were down regulated in the ESET knockout brains, hinting toward an indirect regulation by ESET. Therefore, the methyltransferase ESET is involved in the cell fate regulation of the embryonic NSPCs, by actively suppressing astrogliogenesis.

The H3K9 methyltransferase G9a and the related molecule GLP, appear to function as a constitutive heteromeric complex to promote H3K9 dimethylation in euchromatic regions leading to transcriptional repression (Shinkai and Tachibana, 2011). During the development of the mammalian retina, G9a has been shown to repress progenitor gene expression, which facilitates the terminal differentiation of these cells (Katoh et al., 2012). Indeed, postnatal conditional loss of function of the G9a/GLP complex causes the de-repression of neural progenitor cell genes, as well as non-neural genes, in mature neurons resulting in severe behavioral defects in these animals (Schaefer et al., 2009). It remains to be determined how the G9a/GLP complex is recruited to specific loci to promote transcriptional repression, but such a mechanism would likely be active during the transition from a proliferating progenitor cell to a post-mitotic differentiated neuron or glial cell, and may even persist to ensure the maintenance of repression marks on these progenitor genes in mature cells.

#### Polycomb family members

Additional evidence for the importance of histone methylation on the onset of neurogenesis arises from seminal work on Bmi-1, associated with the Polycomb family of H3K27 methyltransferases (Schuettengruber et al., 2007). Bmi-1^−∕−^ mice exhibit a variety of neurological defects starting from an early age (van der Lugt et al., 1994). Molofsky et al. (2003) showed that Bmi-1^−∕−^ mice demonstrate decreased self-renewal and proliferation in both embryonic and postnatal forebrain NSCs. Bmi-1 enhanced NSC self-renewal and proliferation partly by blocking p16^*Ink*4*a*^ and p19^*Arf*^, 2 inhibitors of cyclin-dependent kinases (Jacobs et al., 1999; Molofsky et al., 2003). Interestingly, Bmi-1 was required for the proliferation of NSCs, but not for that of committed neurogenic or gliogenic progenitors, revealing a regulatory role upstream in the hierarchy of neurogenesis.

In a different report Fasano et al. (2007) utilized a lentivirus-delivered shRNA approach to knockdown the expression of Bmi-1 in embryonic, perinatal or adult NSCs and their results were slightly different. Even though they did observe a decrease in NSC proliferation and self-renewal, the effects of the Bmi-1 knockdown were more pronounced as the age of the mice was increasing. Therefore, adult NSCs seemed to be more dependent on Bmi-1 than embryonic NSCs. The authors attributed their effects to p21 up-regulation upon Bmi-1 knockdown, and when p21 was also knocked down the phenotype was rescued. Unlike Molofsky et al., Fasano et al., did not observe a p16^Ink4a^ or p19^Arf^ up-regulation after Bmi-1 knockdown, and simultaneous knockdown of Bmi-1, p16^Ink4a^ and p19^Arf^ failed to rescue the Bmi-1 phenotype.

The differences between the 2 studies can be attributed to the fact that the knockout mice used by Molofsky et al. might have had compensatory mechanisms in place since they had to develop without Bmi-1, while the shRNA approach provides a more acute decrease in Bmi-1. It is also plausible that the decreased levels of Bmi-1 produced by an shRNA knockdown cannot recapitulate the true null phenotype of a Bmi^−∕−^ mouse, and the presence of a remaining minimal amount of protein is sufficient to alter the observed effects. Additionally, the different mouse backgrounds used in the 2 studies could have also contributed to the observed differences.

**Figure 3:**
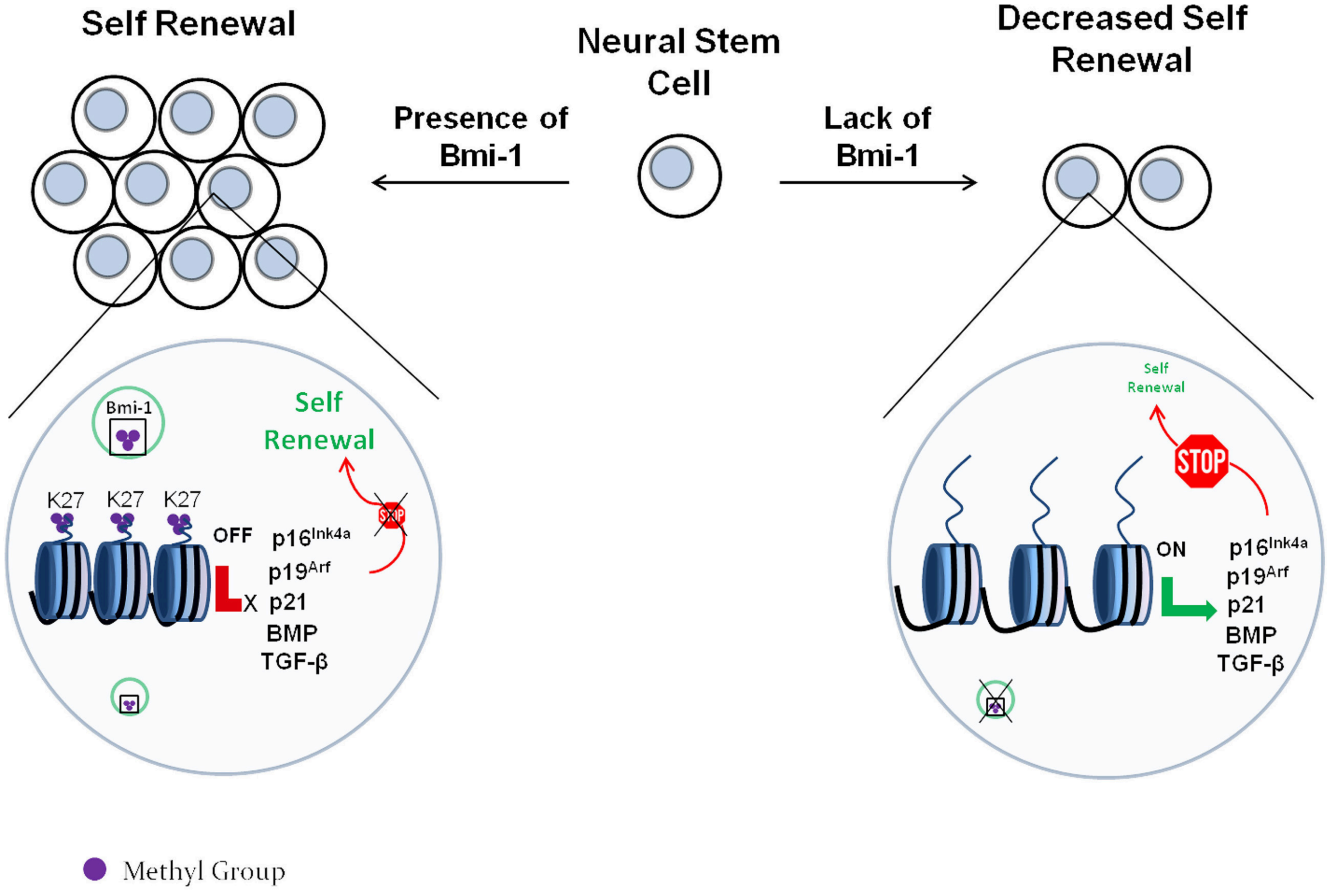
**The role of the Polycomb protein Bmi-1 in neural stem cell self-renewal**. The Polycomb complex trimethylates the regulatory regions of the cdk inhibitors p16^Ink4a^, p19^Arf^, and p21 at the lysine 27 residue of H3. This represses their expression, which allows cell cycle to proceed. In the absence of Bmi-1, the cdk inhibitors are expressed and block the cell cycle. This decreases the self-renewal capability of the neural stem cells.

In a recent report by Gargiulo et al. (2013), Bmi-1 was found by ChIP/RNA-seq to target p16^Ink4a^, p19^Arf^, and p21, along with an array of additional cdk inhibitors. Gargiulo et al. also found that Bmi-1 down-regulates multiple members of the BMP and TGF-β pathways, which are long established to be involved in inducing differentiation of NSCs into neurons or astrocytes (Bond et al., 2012; Rodriguez-Martínez and Velasco, 2012). Therefore, it appears that Bmi-1 enhances the NSC proliferation and renewal by 2 mechanisms: (i) it suppresses cdk inhibitors, thus allowing the NSCs to continue cycling and (ii) it suppresses neurogenic pathways, thus delaying the onset of differentiation. A schematic demonstration of this model is shown in Figure 3.

The converse experiment, namely, over-expression of Bmi-1, resulted in an increase in the self-renewal and proliferation of both embryonic and adult NSCs (Fasano et al., 2009). In addition, the authors showed that FoxG1, a forebrain-specific transcription factor (Shen et al., 2006) was necessary for the Bmi-1—induced increase in NSC self-renewal. This raises the possibility that FoxG1 is a downstream effector of Bmi-1, even though over-expression of FoxG1 was less effective at promoting an increase in NSC self-renewal compared to Bmi-1 over-expression. Therefore, either Bmi-1 utilizes additional downstream effectors, or Bmi-1 and FoxG1 operate in different pathways. Intriguingly, NSC self-renewal was unchanged when Bmi-1 was over-expressed in embryonic spinal cord NSCs, which constitutes additional evidence for the notion that the epigenetic regulation of neurogenesis utilizes divergent pathways in a region-specific manner.

#### Trithorax family members

In addition to the Polycomb family of transcriptional repressors, there exists the Trithorax family of transcriptional activators that operate by methylating H3K4 residues (Schuettengruber et al., 2007). The Trithorax family member Mll1 (Mixed lineage leukemia 1), an H3K4 methyltransferase, was reported to be involved in postnatal neurogenesis in a very interesting manner (Lim et al., 2009). Mll1 seemed to be necessary for the induction of Dlx2, an indispensable transcription factor for olfactory bulb neuron specification and migration (Long et al., 2007). NSPCs isolated from the forebrain of postnatal mice in which Mll1 had been conditionally knocked out survived and proliferated normally, but gave rise to significantly less neurons than controls. Gliogenesis was not affected. Similar phenotypes were observed *in vivo*. Therefore, Mll1 is required specifically for neuronal differentiation of forebrain NSPCs, unlike other epigenetic regulators that we reviewed above that are required for differentiation into all 3 lineages. Surprisingly, Mll1 did not act by catalyzing an H3K4 methylation event, but rather by recruiting an undefined H3K27 demethylase at the Dlx2 promoter. Since H3K27 methylation is inhibitory for gene expression, such H3K27 demethylase would activate transcription. Therefore, just as has been observed in other systems (Schuettengruber et al., 2007), it appears that members of the conserved Polycomb and Trithorax families of methyl-transferases co-operate to regulate neurogenesis. The Polycomb member Bmi-1 ensures that the NSCs remain in cycle for as long as needed to generate enough progeny and then passes the baton to the Trithorax member Mll1 which overviews neuronal differentiation. How this strict temporal pattern of activity of these epigenetic factors is controlled remains to be elucidated.

#### Histone demethylases

An independent report by Jepsen et al. (2007) showed that JMJD3/KDM6B, a putative histone demethylase, steers interneuron differentiation in NSPCs from the mouse embryonic cortex. JMJD3/KDM6B contains a Jumonji C domain, which is responsible for its histone demethylase activity (Klose et al., 2006). A mutant JMJD3/KDM6B lacking this domain was unable to induce interneuron differentiation. Through an *in vitro* demethylation assay, it was shown that JMJD3/KDM6B specifically demethylates H3K27me3, therefore causing a de-repression of the target genes, many of which appear to be neuronal. Treatment of NSCs with retinoic acid, a known inducer of neuronal differentiation (Ribes et al., 2006), induced binding of JMJD3/KDM6B to the Dlx5 promoter and reduction in the H3K27me3 levels as evidenced by ChIP. Dlx5 is expressed in differentiated interneurons (Anderson et al., 1997). JMJD3/KDM6B is normally suppressed by Nuclear Co-repressor 2 (NcoR2 or SMRT), a protein involved in a multitude of developmental processes (Mottis et al., 2013). Knocking out SMRT resulted in significant alterations in the forebrain of mouse embryos as well as decreased proliferation and increased differentiation of embryonic cortex NSPCs into neurons and astrocytes. RA signaling was necessary for the increased neuronal differentiation of the SMRT^−∕−^ cells and JMJD3/KDM6B was significantly up regulated upon the knockout. More recently, it has been demonstrated that JMJD3/KDM6B is actually required for proper neurogenesis (Park et al., 2014). Whether JMJD3/KDM6B is the same H3K27 demethylase that is recruited by Mll1 in the study of Lim et al. is unknown and will require further studies, but it has been shown in other cellular contexts that JMJD3/KDM6B and Mll1 indeed interact (Shi et al., 2014).

In addition to JMJD3/KDM6B, recent evidence indicates that histone demethylases JMJD2A/KDM4A and JMJD2C/KDM4C are important PTM enzymes that contribute to neurogenesis (Cascante et al., 2014). Cascante et al. demonstrated that these enzymes are required for demethylating BDNF regulatory regions at H3K9 in response to the HDAC inhibitor VPA, thus inducing its expression. At the same time, these enzymes are required for demethylation of GFAP exonic regions at H3K36, leading to a blockade of astrogliogenesis. How this differential specificity for methylated lysine is achieved remains to be identified and may involve different cofactors for each promoter. The authors showed that knockdown of JMJD2A/KDM4A and JMJD2C/KDM4C by siRNA in embryonic cortical NSPCs led to precocious astrogliogenesis and decreased neurogenesis associated with increased cell death. These data support previous results from analyses of a different histone demethylase, namely, JHDM1D. Huang et al. (2010) demonstrated that JHDM1D is necessary for neural specification of ES cells, by demethylating regulatory regions of FGF4. Similar to JMJD2A/C, this enzyme appears to be bi-specific: it removes methyl residues from both H3K9 and H3K27.

In conclusion, it appears that the neuronal specification of an NSPC in addition to methyl transferase activities requires the presence of multiple histone demethylases that de-repress neuronal commitment genes that were silent in the progenitors, while at the same time suppressing alternative fates.

#### Histone arginine methylation

Apart from histone methylation on lysine residues, histone methylation on arginine residues has also been implicated in the regulation of neurogenesis. In general, symmetric dimethylation (symbolized as me2s) of an arginine residue is associated with transcriptional repression of the respective gene, while asymmetric dimethylation (me2a) is associated with transcriptional activation (Xu et al., 2010; Di Lorenzo and Bedford, 2011; Chittka et al., 2012). Chittka et al. (2012) reported that the transcription factor Schwann cell factor 1 (SC1, also known as PRDM4) controls the onset of neurogenesis from embryonic NSPCs by recruiting the histone arginine methyltransferase PRMT5. In embryonic NSPCs cultured *in vitro*, SC1 was only expressed in non-terminally differentiated mitotically active progenitors, but not in TuJ1^+^ or GFAP^+^ cells. The levels of SC1 staining decreased over time in culture, along with the increase in differentiation. Silencing of SC1 by siRNA induced precocious neuronal differentiation, and decreased BrdU incorporation, while over-expression of SC1 increased the proportion of Nestin positive cells in the embryonic NSPC cultures. Through a series of immunoprecipitation experiments, the authors showed that SC1 interacts with the methyltransferase PRMT5 through its N-terminal domain. The presence of the N-terminus of SC1 was necessary to achieve the increase in Nestin^+^ cells after over-expression in neural stem cell cultures, indicating that PRMT5 may be necessary for the effect. Additionally, both embryonic NSCs and sections of E10.5 cortex, showed high levels of expression of both PRMT5 and SC1 and very frequent H4R3me2s modifications. The latter is the symmetrical di-methylation catalyzed by PRMT5. The authors also showed that over-expression of SC1 containing the N-terminus, led to a down regulation in the expression of the promitotic genes Bub1b and cyclinB. Conversely, siRNA knockdown of SC1 led to an up-regulation of Bub1b and cyclinB. High levels of promitotic genes have been shown to be required for proper asymmetric division (Tio et al., 2001), raising the possibility that SC1 delays the onset of neurogenesis by impeding asymmetric division of neural stem cells which would give rise to committed progenitors. Nevertheless, more research is needed to directly demonstrate that PRMT5 is necessary for these effects. Interestingly, differentiated neurons correlated with H4R3me2a marks, while undifferentiated precursors correlated with H4R3me2s marks (Chittka, 2010). Therefore, the methylation status of H4R3 appears to function as a binary switch that changes its position along with neuronal differentiation. Whether the epigenetic switch has a causative role in neuronal differentiation or it is a consequence of that differentiation remains to be elucidated.

#### Elucidating a putative histone methylation code of neurogenesis

Despite the plethora of convincing evidence demonstrating that histone methylation is involved in neurogenesis, correlating specific methylation marks with gene expression or repression is still difficult. This complexity was demonstrated in a report by Popova et al. (2012). The authors monitored the presence of H3K4me2 and H3K27me3 during pre- and postnatal retinal development and attempted to correlate that with the expression level of individual genes. No simple correlation was identified as for some genes increases in expression correlated with increased H3K4me2 and decreased H3K27me3, while for others they did not. This demonstrates that the particular epigenetic marks cannot be universally used as predictors of gene expression or repression, and that the specific histone modifications that regulate expression vary between genes. Interestingly, the genes with similar correlations between expression and presence of the 2 epigenetic marks could be grouped with gene ontology. Additionally, the authors were able to identify a specific epigenetic signature that could predict the identity of a gene as photoreceptor-specific. Thus, specific gene families may share common epigenetic mechanisms of expression regulation, and the identification of these mechanisms could generate novel, sensitive genetic tools for screening of genes associated with the development of specific systems.

#### Combining histone acetylation and methylation: the example of CtBPs

We have reviewed histone acetylation and histone methylation as distinct processes; however, there are examples of proteins that combine these 2 activities. C-terminal binding proteins (CtBPs) repress transcription by recruiting both HDAC and H3K9 methyltransferase activities to the promoters (Hildebrand and Soriano, 2002; Shi et al., 2003). Dias et al. (2014) demonstrated that CtBPs are indispensable for repressing neurogenesis in the roof plate (RP), a BMP-secreting structure which is responsible for dorsoventral patterning of neural cells within the neural tube (Liem et al., 1995, 1997; Lee et al., 2000). The authors demonstrated that there is an oxygen gradient within the neural tube, with the RP receiving higher oxygen levels than the neurogenic regions. This leads to higher concentrations of NADH, the reduced form of NAD^+^, within the RP region. NADH interacts with CtBPs (Kim et al., 2005), increasing their transcriptional repressive function. This leads to CtBPs repressing the expression of Math1, a proneural transcription factor, specifically in the higher oxygen environment of the RP, so as to prevent this region from generating neural tissue. Interestingly, CtBPs also bound promoter elements of Hes1, which inhibits neurogenesis, following an inverse correlation with oxygen levels: CtBPs repressed Hes1 expression in the lower oxygen environment of the neural tube. At higher oxygen levels, CtBPs are released from the Hes1 promoter, which enables a blockade of neurogenesis in the RP.

CtBPs have also been shown to be involved in neurogenesis by inhibiting Notch signaling targets in the absence of Notch ligand (Oswald et al., 2005). Data from *Xenopus laevis* and mammalian cell lines have shown that the transcriptional repressor SHARP is required for proper inactivation of Notch signaling (Garriga-Canut et al., 2001; Oswald et al., 2002, 2005). It has been proposed that SHARP blocks transcription by two independent mechanisms, one of which is sensitive to HDAC inhibitors (Garriga-Canut et al., 2001). It was eventually discovered that SHARP interacts with CtBPs and that this interaction is necessary for its ability to suppress Notch signaling and promote differentiation (Oswald et al., 2005). Therefore, CtBP complexes are multimodal histone modifiers that appear to be involved in neurogenesis through diverse pathways.

## Specific considerations

### Initiation of chromatin modifications in the context of neurogenesis

What is it that triggers the initiation of chromatin modifications associated with neurogenesis? It appears that known signals that induce neurogenesis under certain contexts, such as FGFs (Kengaku and Okamoto, 1993), neurotrophins (Bartkowska et al., 2007), bone morphogenic proteins (BMPs) (Andersson et al., 2011; Bond et al., 2012) and other members of the transforming growth factor β (TGFβ) (Rodriguez-Martínez and Velasco, 2012) family also trigger the initiation of histone PTMs. In the example of CREB that was reviewed above, activation is controlled by phosphorylation of serine-133. CREB can be activated by a variety of cellular pathways, including the cAMP pathway, the PI3 kinase pathway, the MAP kinase (MAPK) pathway, or even in response to increased intracellular Ca^2+^ (Dworkin and Mantamadiotis, 2010). Therefore, an array of signaling molecules involved in neurogenesis also leads to CREB activation. Nerve growth factor (NGF), platelet-derived growth factor (PDGF) and FGFs are just some examples of factors that activate the PI3 kinase pathway (Kapeller and Cantley, 1994; Brader and Eccles, 2004). Similarly, MAPK signaling is activated by FGFs (Thisse and Thisse, 2005) and insulin-like growth factor 1 (IGF-1) (Bateman and McNeill, 2006) among others. Thus, a functional link between secreted signals involved in neurogenesis and CREB activation can be established. Additionally, the fact that CREB can be activated in response to elevated intracellular Ca^2+^ and cAMP provides a link between mature neuronal activity and CREB recruitment to chromatin. Interestingly, CREB activation by Ca^2+^ (and not the other pathways) leads to the phosphorylation of 2 additional serine residues, which may alter its target specificity (Kornhauser et al., 2002). In an alternate example, Estarás et al. (2012) showed that Smad3 recruits JMJD3/KDM6B to neuronal promoters in NSCs. This establishes a connection between JMJD3/KDM6B H3K27 demethylase activity and the TGFβ pathway.

It appears therefore, that known exogenous neurogenic signals are sufficient to induce post-translational modifications of transcription factors, which leads to recruitment of histone modifying factors to the chromatin, and thereby changes in chromatin charge and conformation associated with increased gene expression of neurogenic factors. This functions as an additional argument for the notion that chromatin modifications are an essential component of the mechanisms regulating neurogenesis.

### Tissue specific effects of global regulators

Most histone PTM regulators may function globally in the genome. An obvious question that arises from that observation is how a master regulator can perform a tissue specific function. There are several ways that such neurogenesis-specific function of factors with assumed general functions can be achieved. As described in the example above, exogenous signals can influence ubiquitously expressed transcription factors in specific ways resulting in signaling-context-specific recruitment of histone modifying factors to localized regions of the chromatin. In addition, the signature of the pathway that activates a global regulator can be “stamped” on it via specific modifications, which can affect the selection of the downstream targets.

Exogenous signals may also affect the levels of histone modifying factors in the cell. For example, JMJD3/KDM6B expression is highly regulated by retinoic acid, and thus factors with general function may not always be expressed evenly in different cell types, including progenitors. In fact, many factors with general function, including CBP, HDACs, HDMs etc., may not always be ubiquitously expressed and tissue- and cell-specific expression of variants of chromatin-modifying factors have been reported (for example neuro-specific BAFs, Olave et al., 2002).

**Figure 4:**
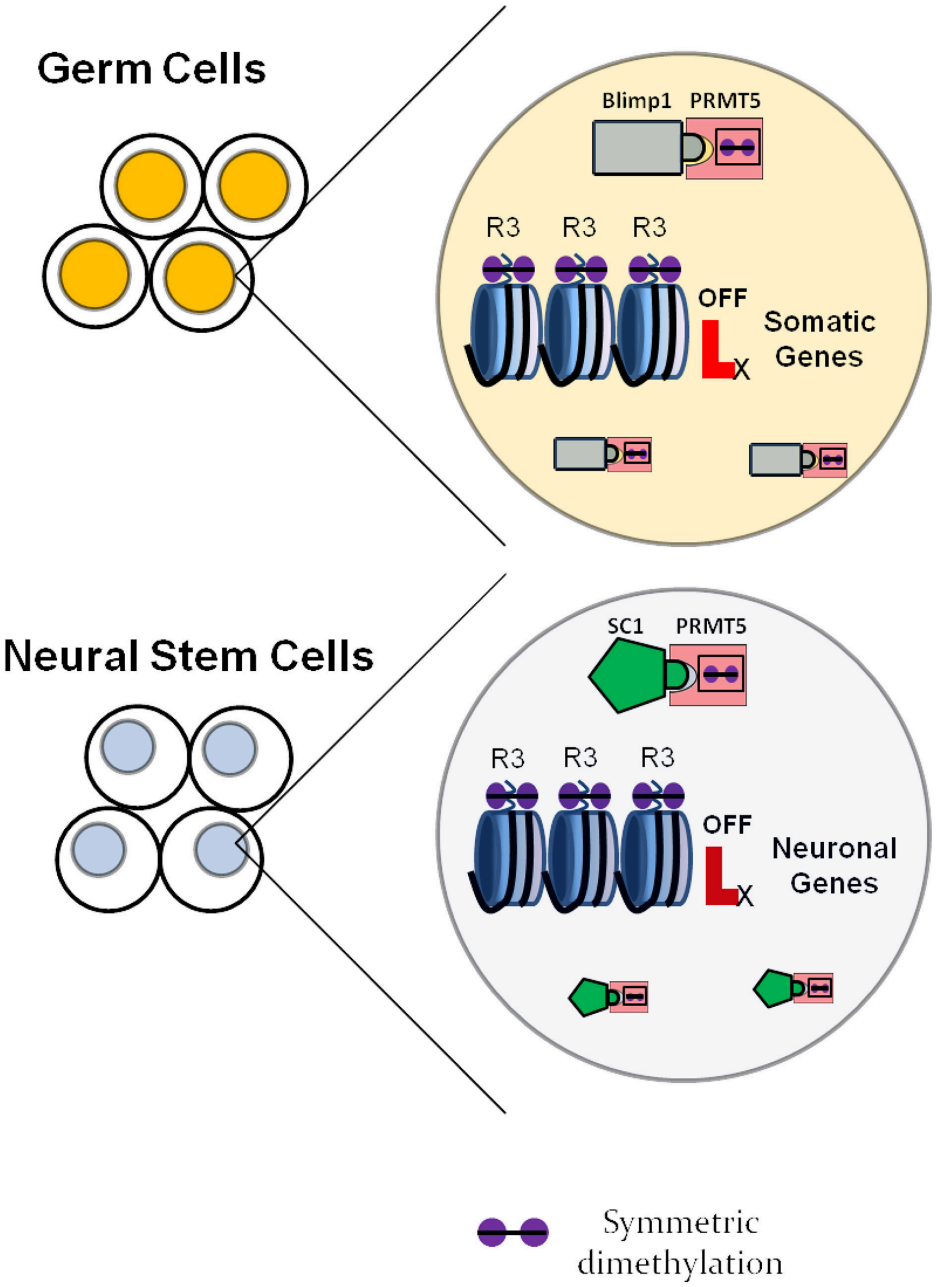
**Neural specific roles of universally expressed histone modulators as exemplified by the arginine methyltransferase PRMT5**. In germ cells, PRMT5 forms a complex with the transcription factor Blimp1 and symmetrically dimethylates regulatory regions of somatic genes, which inhibits their expression. This ensures maintenance of the germ cell fate. In neural stem cells, PRMT5 forms a complex with the transcription factor SC1 and suppresses the expression of neuronal genes, which delays the onset of differentiation and maintains the neural stem cell state.

It is further necessary to consider the combination of factors expressed in a certain cell, as factors with similar function in different cell types may utilize cell-specific adaptors and/or chaperones. Such strategies are utilized by, for example, the arginine methyltransferase PRMT5 (reviewed above), that has been involved in various systems in addition to neurogenesis, such as primordial germ cells, embryonic stem cells and erythrocyte progenitors (Ancelin et al., 2006; Saitou, 2009; Zhao et al., 2009; Xu et al., 2010), always having the function of maintaining cells in an undifferentiated state. In this case, the global gene expression regulator gets recruited within specific cell types by different adaptor proteins. For neural stem cells, the adaptor protein seems to be the transcription factor SC1, while for primordial germ cells the adaptor protein is known to be Blimp1 (Ancelin et al., 2006), as shown in Figure 4.

While these examples appear straightforward, it is also important to keep in mind that there is cross-talk among the histone modifications themselves. As the modifications alter chromatin charge and conformation, they can facilitate or hinder secondary and even tertiary histone modifications and thereby directly influence the recruitment of factors to promote neurogenesis.

### Chromatin modification complexes encompassing “write,” “read,” and “erase” functions, that have a role in neurogenesis

Considering each histone modification enzyme in isolation obscures the true picture of how these proteins are regulated. Usually, chromatin regulation operates through complexes that integrate the many aspects of epigenetics, in addition to histone PTMs.

In the context of neurogenesis, one of the most well established complexes is the one that converges on element-1 silencing transcription factor/neuron-restrictive silencing factor (REST/NRSF) (Qureshi et al., 2010; Jobe et al., 2012). REST is a transcription factor that recognizes the repressor element 1 (RE1) and was long believed to only function as a transcriptional repressor of neuronal genes in non-neural tissues. REST knockouts are embryonic lethal with a range of neural and non-neural malformations. REST can be induced by a variety of signals and has in addition been suggested to induce genes involved in neurogenesis and participate in neuronal differentiation and maturation (Qureshi et al., 2010). Similar to other zinc finger transcription factors, REST functions as a central node that recruits a massive array of epigenetic modifiers. An interesting key to the mechanisms underlying the many functions of REST came with the discovery of an interacting regulatory protein, Co-REST (Andrés et al., 1999). Co-REST is associated with the CtBP complex (see above) and is therefore a primary example of the plethora of enzymes with various functions recruited to DNA and thus chromatin by single transcription factors. These proteins include “writers” such as histone methyltransferases, “erasers” such as histone deacetylases, and “readers” such as methyl-DNA binding proteins. Additionally, REST recruits non-coding RNAs, components of the SWI/SNF remodeling complex and various adaptor proteins with a wide range of activities (Yoo and Crabtree, 2009). Therefore, REST forms signal- and cell-context specific complexes that make use of many of the known epigenetic mechanisms.

Additional examples of such multifunctional epigenetic complexes are the ones formed by the H3K27 methyltransferases of the Polycomb group. We reviewed the role of Bmi-1 in neurogenesis above. Bmi-1 does not operate in isolation, but rather as part of a multi-protein complex called PRC1 (Polycomb Repressive group Complex 1). The complex exists in many alternative forms that contain H3K27me3 binding proteins (“readers”), histone ubiquitin ligases (“writers”), and additional histone binding proteins as well as activity regulators and proteins of unknown function (Luis et al., 2012; Aloia et al., 2013). This enzyme complex can either operate in isolation, or together with a different complex of the Polycomb family, PRC2. PRC2 contains the H3K27 methyltransferase enhancer of zeste 1 or 2 (EZH1 or EZH2, “writers”), along with H3K27me3 binding proteins, additional histone and DNA binding proteins (“readers”) and enzymatic regulators (Margueron and Reinberg, 2011; O'Meara and Simon, 2012). PRC2 also has a pivotal role in neurogenesis, as has been demonstrated by studies on EZH2 and other components of the complex (Pasini et al., 2007; Pereira et al., 2010; Chou et al., 2011). In addition to the core components of the complexes, their recruitment to chromatin is dependent on DNA methylation and non-coding RNAs (Margueron and Reinberg, 2011; Jobe et al., 2012; Aloia et al., 2013). These complexes therefore constitute another example of integration of multiple epigenetic mechanisms within one pathway.

It is becoming increasingly evident that the interactions between different epigenetic modifications play key roles in cellular differentiation, and that the variable composition of many poly-enzymatic complexes is a way of conferring tissue specificity. Additional research in this field will improve our understanding of the epigenetic component of neurogenesis and may reveal novel mechanisms of initiation of chromatin remodeling. Furthermore, how these histone PTMs are sustained for a certain developmental interval without being removed prematurely remains elusive.

### Histone dynamics: an unchartered territory for understanding chromatin regulation of neurogenesis

Even though a great deal of research has focused on the effects of histone PTMs, an interesting direction would be looking into the impact of histone abundance and turnover on neurogenesis. Histones are relatively stable proteins, and their half-life in non-dividing brain cells was found to average 159 days (Commerford et al., 1982). The amount of histones within a cell is tightly regulated, since a histone insufficiency would impede the efficient packaging of DNA and lead to cell death, while an excess of histones can cause chromatin aggregation due to their highly positive charge (Steger and Workman, 1999; Nelson et al., 2002; Ye et al., 2003; Gunjan et al., 2006). The dynamics of histone synthesis differ between diving and non-diving cells, since dividing cells have to couple histone with DNA synthesis at the S-phase (Gunjan et al., 2006). Thus, non-terminally differentiated cells such as NSPCs will synthesize histones at a faster rate than terminally differentiated neurons. Accordingly, a cathepsin-dependent mechanism for histone H3 turnover has been described in differentiating ES cells (Duncan et al., 2008). Additionally, there is evidence that histone turnover differs between actively transcribed and silent regions of the genome, as well as that epigenetic modification can alter histone turnover (Henikoff, 2008; Deal et al., 2010). Most of the effects of histone PTMs in the literature have been assessed within the conceptual framework of stable nucleosomes and do not take into account potential effects on histone dynamics. Indeed, the latter could prove to be a major mechanism of action of epigenetic modifications, especially in light of recent advances in techniques to measure histone turnover (Deal et al., 2010) and findings of aberrant histone turnover in patients with the neurodevelopmental disorder Rett syndrome (Lilja et al., 2013). Whether the histone abundance in and of itself plays a role in cellular differentiation is a consideration that has not been addressed in existing research. It would be interesting to investigate the consequences of enhanced or suppressed histone synthesis on NSPC behavior.

## Conclusion

Histone PTMs are an essential component of both embryonic and adult neurogenesis. Some of them are induced by neurogenic signals and appear to be under the control of specific signaling pathways that are involved in neurogenesis in diverse ways. More research is needed to decipher the exact pathways that trigger histone modification by other enzymes, as well as the mechanisms that lead to the tissue-specific effects of global modulators. It is becoming clear that many of these answers lie in the intricate albeit specific crosstalk between different epigenetic modifiers within and among enzymatic complexes. Future research into how the specific signaling networks influence the chromatin modifiers and their interactions in specific aspects of NSPC differentiation and other cellular events will increase our understanding of the process of neurogenesis and the pathologies underlying neurodevelopmental and psychiatric disorders.

## Author contributions

NM, VT, OH made substantial contributions to the conception of the work; NM wrote the first draft of the manuscript and NM, VT, OH revised it critically for important intellectual content; NM, VT, OH gave final approval of the version to be published and agreed to be accountable for all aspects of the work in ensuring that questions related to the accuracy or integrity of any part of the work are appropriately investigated and resolved.

## Funding

Funding in the Tropepe Lab is supported by CIHR. Funding in the Hermanson Lab is supported by VR-MH, BCF, and CF. NM is the recipient of an IBBME International Scholar's Program award and an NSERC Create in m3 award.

### Conflict of interest statement

The authors declare that the research was conducted in the absence of any commercial or financial relationships that could be construed as a potential conflict of interest.
